# Biomechanical study of the stability of posterior cervical expansive open-door laminoplasty combined with bilateral C4/5 foraminotomy and short-segment lateral mass screw fixation: a finite element analysis

**DOI:** 10.1186/s13018-024-05050-x

**Published:** 2024-10-03

**Authors:** Kunpeng Li, Qun Yu, Chongyi Wang, Runtong Zhang, Qingyang Fu, Yunze Feng, Chen Liu, Xinlong Wang, Ronghan Zhang, Le Li, Haipeng Si

**Affiliations:** 1 Department of Orthopedics, Qilu Hospital, Shandong University, Jinan, Shandong 250012 China; 2 Key Laboratory of Qingdao in Medicine and Engineering, Department of Orthopedics, Qilu Hospital (Qingdao), Shandong University, Qingdao, Shandong 266035 China; 3 School of Physics and Electronic Science, Shandong Normal University, Jinan, Shandong 250014 China

**Keywords:** Biomechanics, Posterior cervical expansive open-door laminoplasty, Lateral mass screw, Bilateral C4/5 foraminotomy, Finite element analysis

## Abstract

**Background:**

Posterior cervical expansive open-door laminoplasty (EODL) may cause postoperative C5 palsy, and it can be avoided by EODL with bilateral C4/5 foraminotomy. However, prophylactic C4/5 foraminotomy can compromise cervical spine stability. To prevent postoperative C5 palsy and boost cervical stability, We propose a new operation method: EODL combined with bilateral C4/5 foraminotomy and short-segment lateral mass screw fixation. However, there are no studies on the biomechanical properties of this surgery.

**Purpose:**

Evaluating the biomechanical characteristics of EODL combined with bilateral C4/5 foraminotomy and short-segment lateral mass screw fixation and other three classic surgery.

**Methods:**

An original model (A) and four surgical models (B-E) of the C2-T1 vertebrae of a female patient were constructed. (B) EODL; (C) EODL combined with bilateral C4/5 foraminotomy; (D) C3-6 expansive open-door laminoplasty combined with bilateral C4/5 foraminotomy and short-segment lateral mass screw fixation; (E) C3-6 expansive open-door laminoplasty combined with bilateral C4/5 foraminotomy and C3-6 lateral mass screw system. To compare the biomechanical properties of cervical posterior internal fixation; (E) C3-6 expansive open-door laminoplasty combined with bilateral C4/5 foraminotomy and C3-6 lateral mass screw system. To compare the biomechanical properties of cervical posterior internal fixation methods, six physiological motion states were simulated for the five models using a 100N load force and 1.5Nm torque. The biomechanical advantages of the four internal fixation systems were evaluated by comparing the ranges of motion (ROMs) and maximum stresses.

**Results:**

The overall ROM of Model C outperformed the other four models, reaching a maximum ROM in the extension state of 10.59°±0.04°. Model C showed a significantly higher ROMs of C4/5 segment than other four models. Model D showed a significantly lower ROM of C4/5 segment than both Model B and Model C. Model E showed a significantly lower ROM of C4/5 segment than Model D. The stress in the four surgical models were mainly concentrated on the internal fixation systems.

**Conclusion:**

EODL combined with bilateral C4/5 foraminotomy and short-segment lateral mass screw fixation can maintain the stability of the spine and has minimal effects on the patient’s cervical spine ROMs in the extension and flexion state. As a result, it may be a promising treatment option for cervical spondylotic myelopathy (CSM) to prevention of postoperative C5 palsy.

## Introduction

Cervical spondylotic myelopathy (CSM) is a common clinical degenerative condition of the cervical spine that often needs surgical intervention [1–3]. The primary surgical procedure for treating CSM is posterior cervical expansive open-door laminoplasty (EODL). However, this procedure is prone to various postoperative complications, with C5 palsy being the predominant short-term issue. The long-term complications include cervical spine instability and axial symptoms [4–6]. There are two main hypotheses regarding the etiology of C5 nerve root palsy after EODL [7]: One hypothesis thought that C5 nerve root palsy is mainly associated with nerve root tethering effect due to posterior displacement of the spinal cord after EODL. Another hypothesis is that C5 nerve root palsy is caused by acute decompression and expansion of the spinal cord against chronic compressive diseases. Based on the first hypothesis, Keiichi Katsumi [7] combined prophylactic bilateral C4/5 foraminotomy with EODL to prevent postoperative C5 palsy in a prospective study. The results show that Prophylactic bilateral C4/C5 foraminotomy significantly decreased the incidence of postoperative C5 palsy. However, prophylactic bilateral C4/5 foraminotomy is prone to articular synapse damage. It can compromise cervical spine stability and is likely to leave patients with long-lasting neck pain [8]. In contrast, EODL combined with short segmental lateral mass screw fixation can help preserve the curvature of the cervical vertebrae and boost cervical stability [9]. To prevent postoperative C5 palsy and boost cervical stability, we proposed a new surgery method: EODL combined with bilateral C4/5 foraminotomy and short-segment lateral mass screw fixation.

However, there are no related biomechanics research about EODL with bilateral C4/5 foraminotomy and short segmental lateral mass screw fixation. In this study, we established three-dimensional (3D) finite element(FE) model of EODL with bilateral C4/5 foraminotomy and short segmental lateral mass screw fixation to compare the biomechanical stability with other classical surgery method.

## Materials and methods

### Construction of the FE cervical spine model

**Fig. 1:**
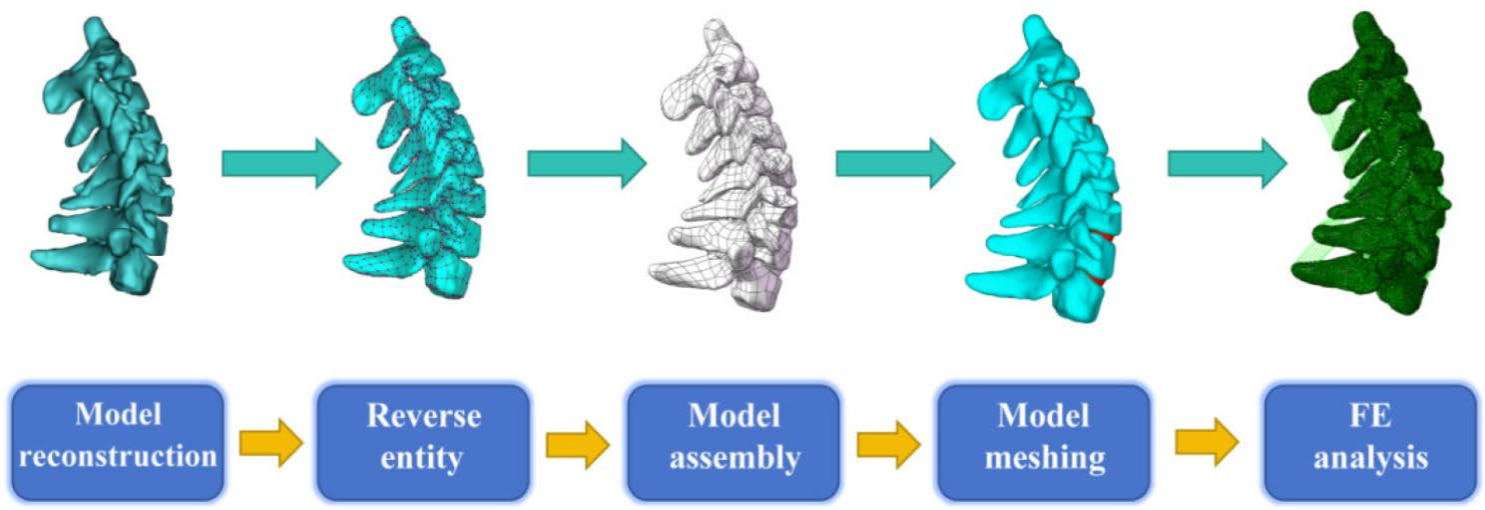
The finite element modeling procedure

A healthy adult female participant was selected for this study. Computed tomography (CT) scan of the cervical vertebrae was carried out. The scan datas were saved in DICOM format and imported into a medical image processing program Mimics 19.0 (Materialise Technologies, Belgium) to obtain 3D model. The 3D model was then imported into Geomagic Studio 12 (Geomagic, NC, USA) for simplification and smoothing. An intact finite element model of the cervical spine (C2-T1) was established in this study. The model comprises intervertebral discs, cartilage endplates, and vertebrae joints [10]. It was assembled in SolidWorks 2016 (Dassault Systems, USA). The assembled model was then imported into Hypermesh 14.0 (Altair, MI, USA) to for pre-processing. To optimize the calculation resource, the size of the tetrahedral mesh was set to 1 mm, and the meshed vertebrae were then reintroduced into Mimics to carry out material assignments based on CT grey value using empirical formulas [11]. Our 3D FE analysis procedure are represent in Fig. 1. Table 1 provides specifics regarding the composition and characteristics of the meshes [12].

**Table 1:** Material properties of finite element models

Component	Element type	Youngs modulus (MPa)	Poissons ratio	Cross section area (mm 2 )
Annulus fibrosus	C3D4	450	0.3	-
Nucleus pulposus	C3D4	1	0.49	-
End plate	C3D4	1200	0.29	-
Cartilage	C3D4	10.4	0.4	-
Internal fixation system	C3D4	110,000	0.3	-
Anterior longitudinal ligament	T3D2	30	0.3	3.1
Posterior longitudinal ligament	T3D2	20	0.3	5.4
Ligamentum flavum	T3D2	1.5	0.3	50.1
Capsular ligament	T3D2	20	0.3	46.6
Interspinous ligament	T3D2	1.5	0.3	13.1
Supraspinous ligament	T3D2	1.5	0.3	5

### Construction of surgical models

**Fig. 2:**
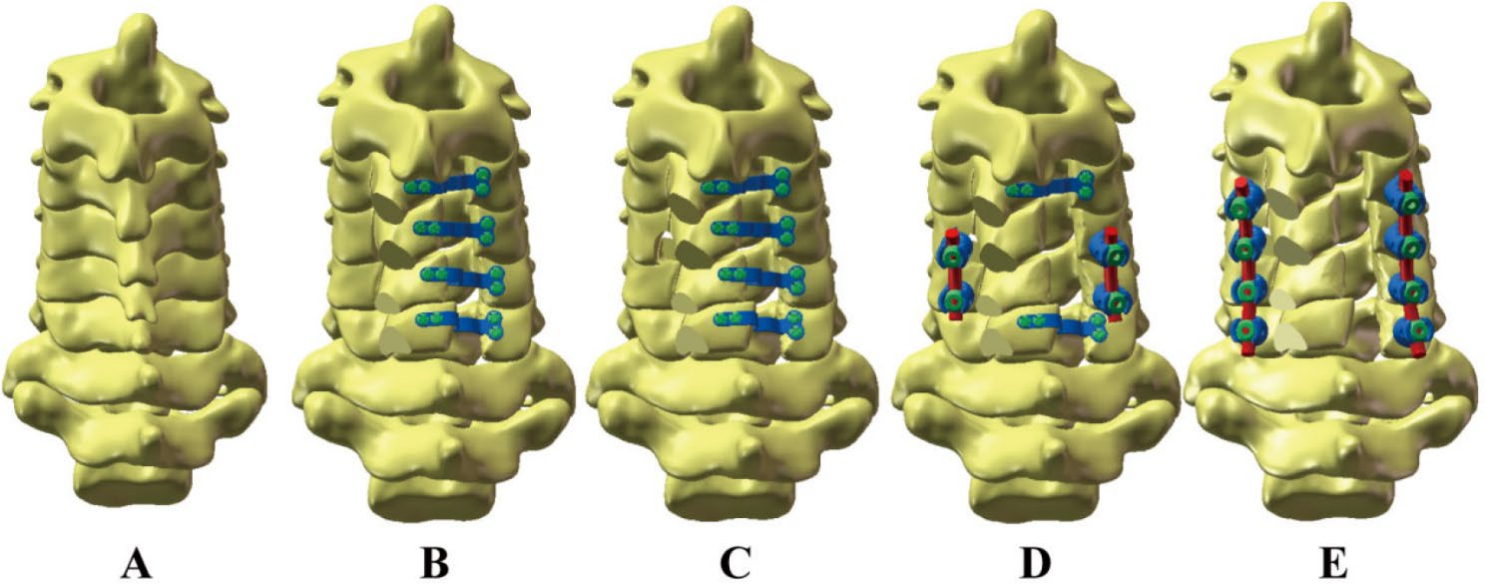
Schematic representation of five finite element models. (**A**) Intact cervical spine; (**B**) Posterior cervical expansive open-door laminoplasty; (**C**) Posterior cervical expansive open-door laminoplasty combined with bilateral C4/5 foraminotomy; (**D**) Posterior cervical expansive open-door laminoplasty combined with bilateral C4/5 foraminotomy and C4/5 lateral mass screw fixation; (**E**) Posterior cervical expansive open-door laminoplasty combined with bilateral C4/5 foraminotomy and C3-6 lateral mass screw fixation

**Fig. 3:**
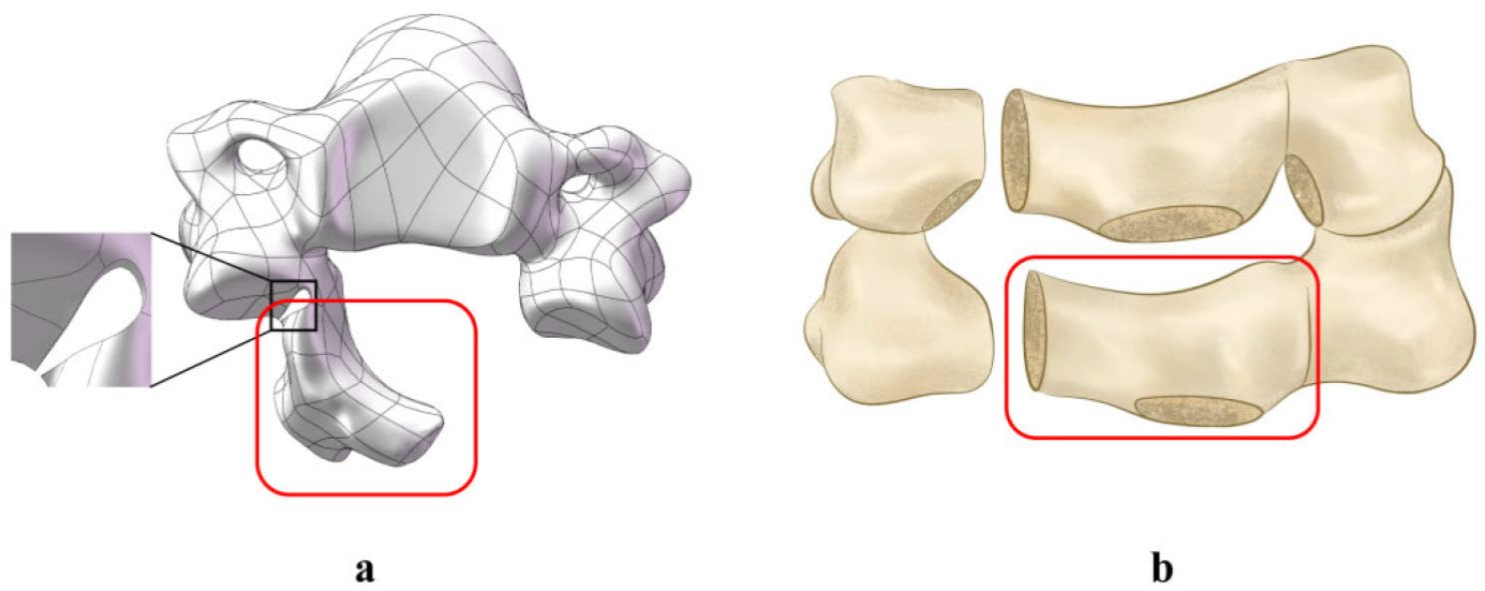
Schematic diagram of posterior cervical spine surgery (**a**) and decompression of C4/5 nerve root canal (**b**) ; The red boxed parts are the vertebral plate

**Fig. 4:**
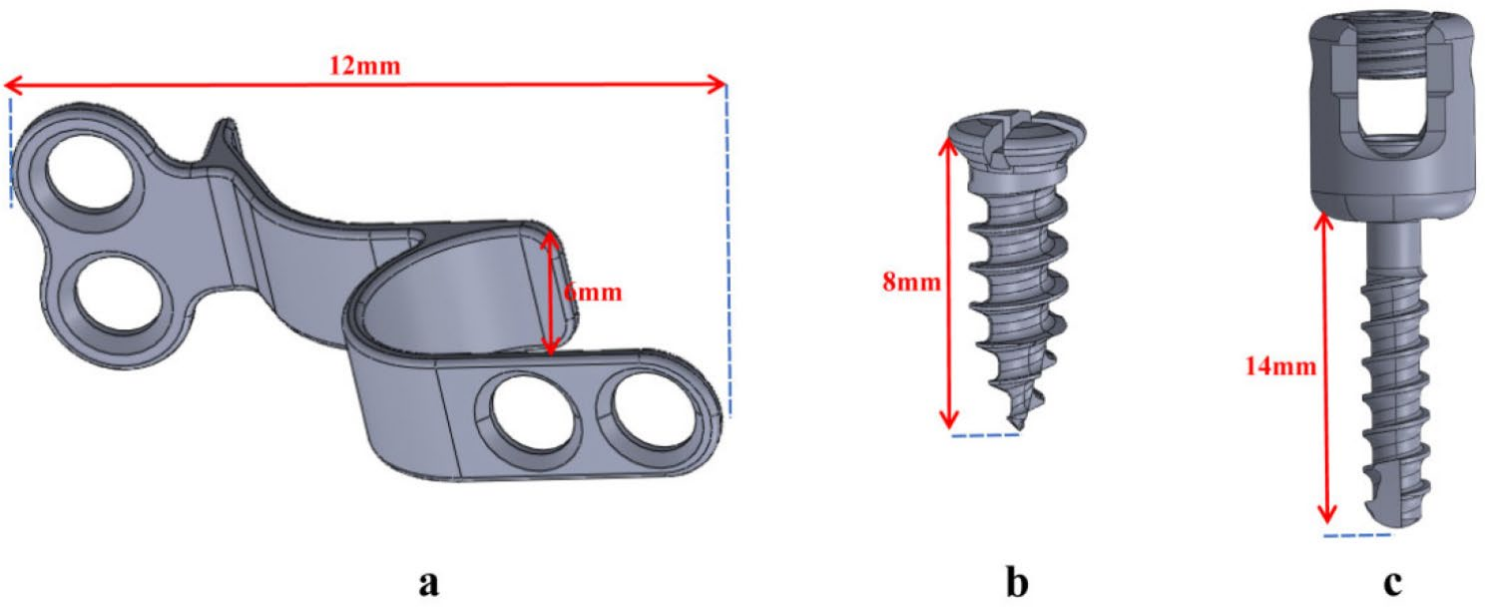
Dimensions of the implants. (**a**) support plate; (**b**) fixing nail; (**c**) lateral mass screw

In this experiment, as shown in Fig. 2, five separate models were set up. Model A simulates an intact cervical spine. Model B represents internal fixation with a C3-6 expanding open-door laminoplasty screw-plate system. Model C shows a bilateral C4/5 foraminotomy combined with a C3-6 expanding open-door laminoplasty screw-plate system. Model D shows C3-6 expansive open-door laminoplasty combined with bilateral C5 foraminotomy fixed by lateral mass screw on C4/5. Model E shows C3-6 expansive open-door laminoplasty combined with bilateral C4/5 foraminotomy and C3-6 lateral mass screw system. Models B-E show the implementation of expansive open-door laminoplasty of the C3-6 segment on the basis of Model A. To perform the “expansive open-door laminoplasty” procedure, the right vertebral plate was first completely removed from the inner edge of the articular process, and the left vertebral plate was partially abraded at the same location (U-shaped groove structure) and then rotated on this axis (Fig. 3a). Models C-E show the “key-hole” procedure involving partially removal of the upper and lower facet at the C4/5 segment. To minimize postoperative facet instability, more than 50% of facet resections should be avoided. We preserves one-half of articular processes on the open side. To prevent hinge side fracture of laminae, we preserve approximately two-thirds of articular processes and laminae cranial to C4 pedicle on the hinge side (Fig. 3b). Screws and steel plates are assembled by moving and twisting them in accordance with the anatomical structure, all of the aforementioned processes are carried out in SolidWorks. Dimensions of the implants are shown in Fig. 4.

### Boundary conditions

The contact type between different bone and implant was set to full constraint [4], whereas the small joints’ contact type was “sliding friction” with a coefficient of friction of 0.3. The lower surface of the T1 vertebral body was completely immobilized, and a load force of 100 N was applied vertically downward to the center of the C2 odontoid to simulate the weight of the skull. A 1.5 Nm torque was also applied to simulate flexion/extension, lateral (right/left) bending, and axial (right/left) rotation [4] .

### Statistical analysis

We counted the range of motion (ROM) of the five models under different motion state conditions, and three sets of points were selected on the upper surface of C2/C3/C5/C5/C6 according to the gradient to mark the activity of the models before and after the motion. The data were analyzed with SPSS 22.0. One-way ANOVA was used for comparisons between several groups, and the independent-sample T test was utilized for comparisons between two groups. Measurement information was expressed as the mean and standard deviation(s). Mathematical ratio calculations were used to compare the angles. *P* < 0.05 was considered statistically significant.

## Results

### The validation of FE models

**Fig. 5:**
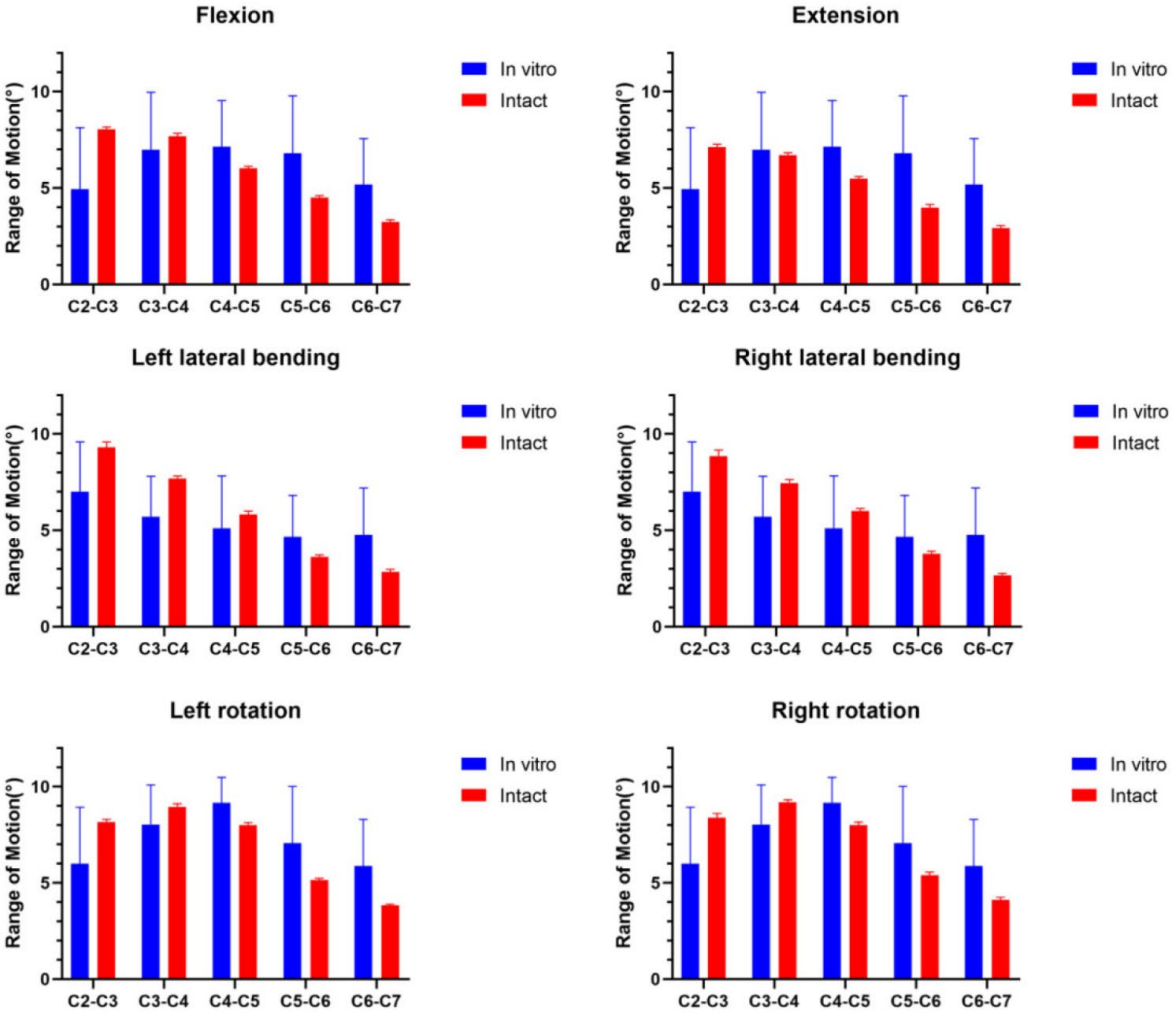
Comparison of the intact model A with other scholars for ROMs

The current model was validated by comparing with the results of other scholars’ models [13]. C2 vertebrae was subjected to a 1.5 Nm torque in Model A, and the range of motion (ROM) in flexion, extension, left and right rotation, and left and right lateral bending was measured. The obtained data were averaged, and the ROMs of Model 1 was depicted in Fig. 5; Table 2. The ROMs of Model A was found to be consistent with the findings of other researchers (*P* > 0.05), demonstrating satisfactory validity.

**Table 2:** Comparison of the intact model A with other scholars for ROMs

ROM(°)		Flexion	Extension	Left lateral bending	Right lateral bending	Left rotation	Right rotation
In vitro	C2/3	4.93 ± 3.20		7.01 ± 2.57		6.00 ± 2.93	
	C3/4	6.98 ± 2.98		5.69 ± 2.11		8.04 ± 2.05	
	C4/5	7.14 ± 2.40		5.09 ± 2.73		9.16 ± 1.32	
	C5/6	6.80 ± 2.99		4.66 ± 2.15		7.06 ± 2.96	
	C6/7	5.17 ± 2.39		4.97 ± 2.42		5.88 ± 2.42	
Intact model	C2/3	7.13	8.05	9.30	8.86	8.17	8.39
	C3/4	6.71	7.68	7.69	7.45	8.95	9.19
	C4/5	5.47	6.02	5.82	6.00	8.02	8.01
	C5/6	3.96	4.50	3.62	3.78	5.13	5.40
	C6/7	2.93	3.22	2.85	2.65	3.79	4.12

### Comparison of the mobility of different FE models

**Fig. 6:**
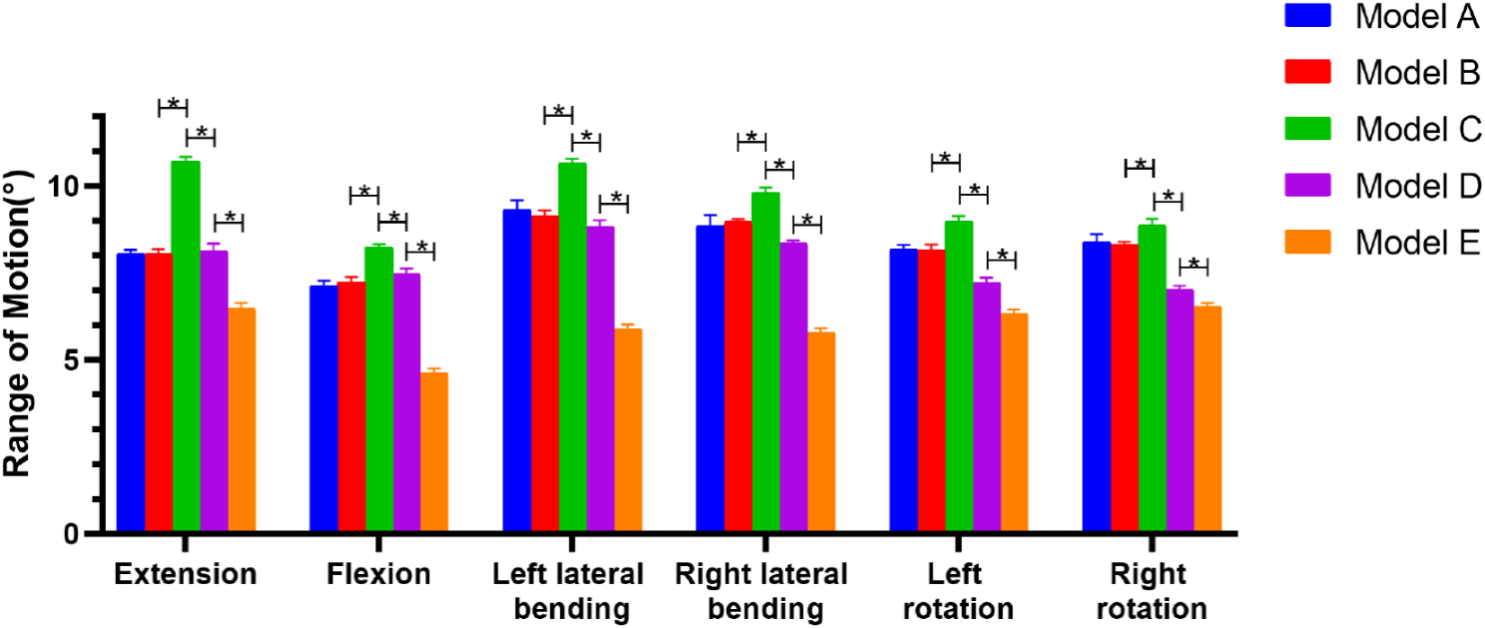
Overall ROMs in different positions in each model. * is indicated above the horizontal bars. * *p* < 0.05

**Fig. 7:**
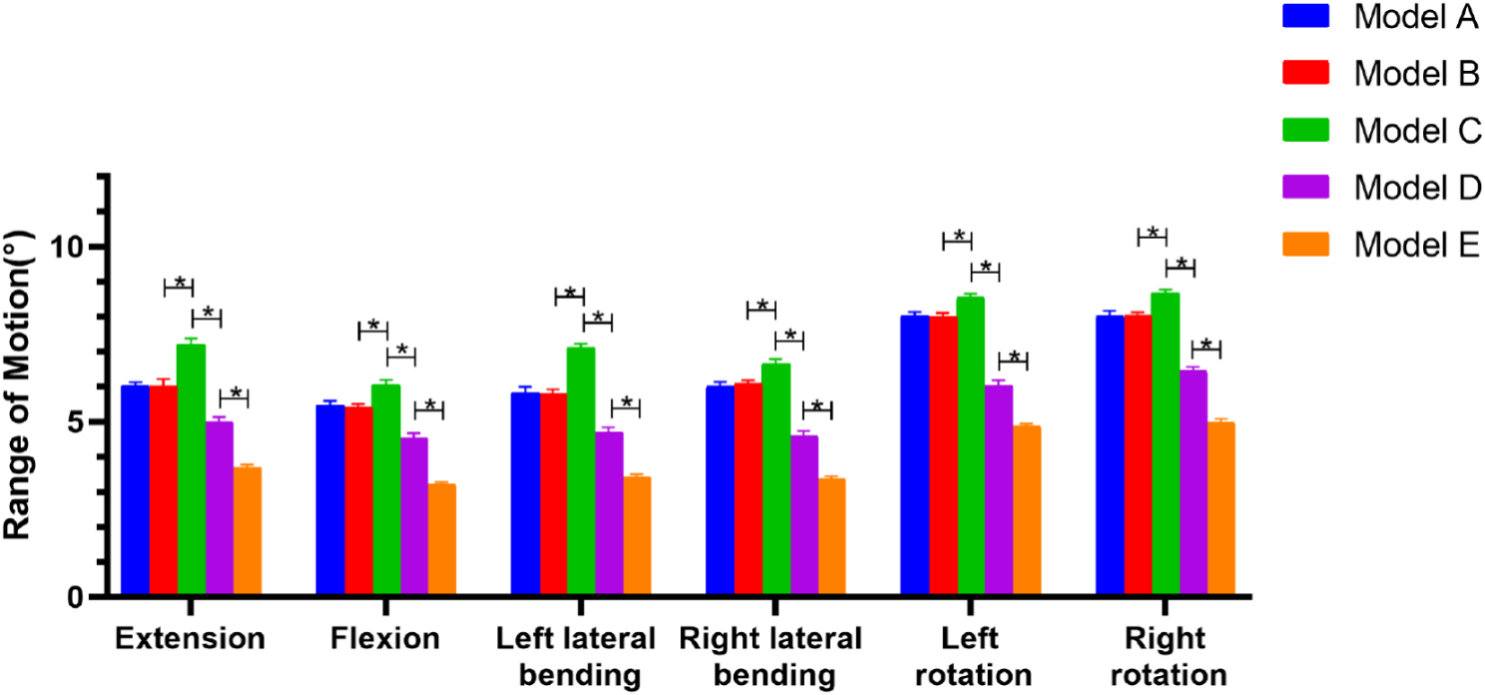
ROM of C4/5 segment in different positions in each model. * is indicated above the horizontal bars. * *p* < 0.05

Model A and Model B did not differ significantly in the six movement states for the overall ROM (Fig. 6). Model C outperformed the other four groups of models in terms of overall ROM, reaching a maximum overall ROM in the extension state of 10.59°±0.04°, with a statistically significant difference (*P* < 0.05). In both flexion and extension, the overall ROM of Model D was somewhat higher than that of Model B. Although there was no statistically significant difference (*P* > 0.05). Model E displayed a statistically significant difference (*P* < 0.05) from the other four groups of models in terms of overall ROM, with a minimum flexion angle of 4.73°±0.03°. The results for the ROM of the C4/5 segments were similar to those for overall mobility (Fig. 7). Model C showed a significantly higher ROM of the C4/5 segment than other surgical models. Model D showed a significantly lower ROM of the C4/5 segment than both Model B and Model C. Model E demonstrated a significantly lower ROM of the C4/5 segment than Model D. Additionally, the ROM of the C3/4, C5/6, and C6/7 segments of Model A-E were examined (Tables 3, 4 and 5).

**Table 3:** ROMs of C3/4 segment in six positions in each model

	Model A a ± b	Model B a ± b	Model C a ± b	Model D a ± b	Model E a ± b	*P* value Model D vs. Model A	*P* value Model D vs. Model B	*P* value Model D vs. Model C
Flexion	7.68 ± 0.17	7.49 ± 0.38	8.76 ± 0.01	6.58 ± 0.03	4.08 ± 0.03	*P* < 0.01^*^	*P* < 0.01^*^	*P* < 0.01^*^
Extension	6.71 ± 0.08	6.01 ± 0.09	7.22 ± 0.03	6.07 ± 0.05	3.38 ± 0.02	*P* < 0.01^*^	*P* < 0.01^*^	*P* < 0.01^*^
Left lateral bending	7.69 ± 0.14	7.59 ± 0.03	8.76 ± 0.03	6.95 ± 0.05	3.60 ± 0.02	*P* < 0.01^*^	*P* < 0.01^*^	*P* < 0.01^*^
Right lateral bending	7.45 ± 0.19	7.22 ± 0.04	8.01 ± 0.03	6.48 ± 0.03	3.66 ± 0.02	*P* < 0.01^*^	*P* < 0.01^*^	*P* < 0.01^*^
Left rotation	8.95 ± 0.16	8.82 ± 0.09	9.54 ± 0.04	8.35 ± 0.03	5.60 ± 0.03	*P* < 0.01^*^	*P* < 0.01^*^	*P* < 0.01^*^
Right rotation	9.19 ± 0.13	9.17 ± 0.16	9.46 ± 0.03	8.53 ± 0.03	5.63 ± 0.03	*P* < 0.01^*^	*P* < 0.01^*^	*P* < 0.01^*^

*P value with the statistical significance; a: mean; b: standard deviation

**Table 4:** ROMs of C5/6 segment in six positions in each model

	Model A a ± b	Model B a ± b	Model C a ± b	Model D a ± b	Model E a ± b	*P* value Model D vs. Model A	*P* value Model D vs. Model B	*P* value Model D vs. Model C
Flexion	4.50 ± 0.10	4.51 ± 0.11	5.13 ± 0.04	4.78 ± 0.02	3.54 ± 0.02	*P* < 0.01^*^	*P* < 0.01^*^	*P* < 0.01^*^
Extension	3.96 ± 0.08	3.97 ± 0.04	4.66 ± 0.06	4.13 ± 0.01	3.12 ± 0.02	*P* < 0.05^*^	*P* < 0.05^*^	*P* < 0.01^*^
Left lateral bending	3.62 ± 0.10	3.63 ± 0.02	4.34 ± 0.04	4.53 ± 0.02	3.39 ± 0.03	*P* < 0.01^*^	*P* < 0.01^*^	*P* < 0.05^*^
Right lateral bending	3.78 ± 0.13	3.70 ± 0.08	4.37 ± 0.03	4.62 ± 0.03	3.33 ± 0.08	*P* < 0.01^*^	*P* < 0.01^*^	*P* < 0.05^*^
Left rotation	5.13 ± 0.11	5.14 ± 0.09	5.74 ± 0.03	5.64 ± 0.03	4.54 ± 0.03	*P* < 0.01^*^	*P* < 0.01^*^	*P* > 0.05
Right rotation	5.40 ± 0.17	5.17 ± 0.05	5.87 ± 0.04	5.74 ± 0.02	4.66 ± 0.04	*P* < 0.01^*^	*P* < 0.01^*^	*P* > 0.05

*P value with the statistical significance; a: mean; b: standard deviation

**Table 5:** ROMs of C6/7 segment in six positions in each model

	Model A a ± b	Model B a ± b	Model C a ± b	Model D a ± b	Model E a ± b	*P* value Model D vs. Model A	*P* value Model D vs. Model B	*P* value Model D vs. Model C
Flexion	3.23 ± 0.06	3.09 ± 0.08	3.70 ± 0.05	3.13 ± 0.04	3.13 ± 0.01	*P* > 0.05	*P* > 0.05	*P* < 0.01^*^
Extension	2.93 ± 0.09	2.86 ± 0.09	3.09 ± 0.04	2.86 ± 0.03	2.91 ± 0.02	*P* > 0.05	*P* > 0.05	*P* < 0.01^*^
Left lateral bending	2.84 ± 0.13	2.71 ± 0.04	3.16 ± 0.05	3.25 ± 0.03	2.54 ± 0.07	*P* < 0.01^*^	*P* < 0.01^*^	*P* > 0.05
Right lateral bending	2.65 ± 0.11	3.73 ± 0.02	3.09 ± 0.04	3.06 ± 0.06	2.58 ± 0.08	*P* < 0.01^*^	*P* < 0.01^*^	*P* > 0.05
Left rotation	3.79 ± 0.10	3.92 ± 0.08	4.12 ± 0.01	4.08 ± 0.05	3.74 ± 0.07	*P* < 0.01^*^	*P* > 0.05	*P* > 0.05
Right rotation	4.12 ± 0.14	4.22 ± 0.07	4.73 ± 0.02	4.50 ± 0.05	4.10 ± 0.03	*P* < 0.01^*^	*P* < 0.01^*^	*P* < 0.05^*^

*P value with the statistical significance; a: mean; b: standard deviation

### Comparison of stresses for different FE models

**Fig. 8:**
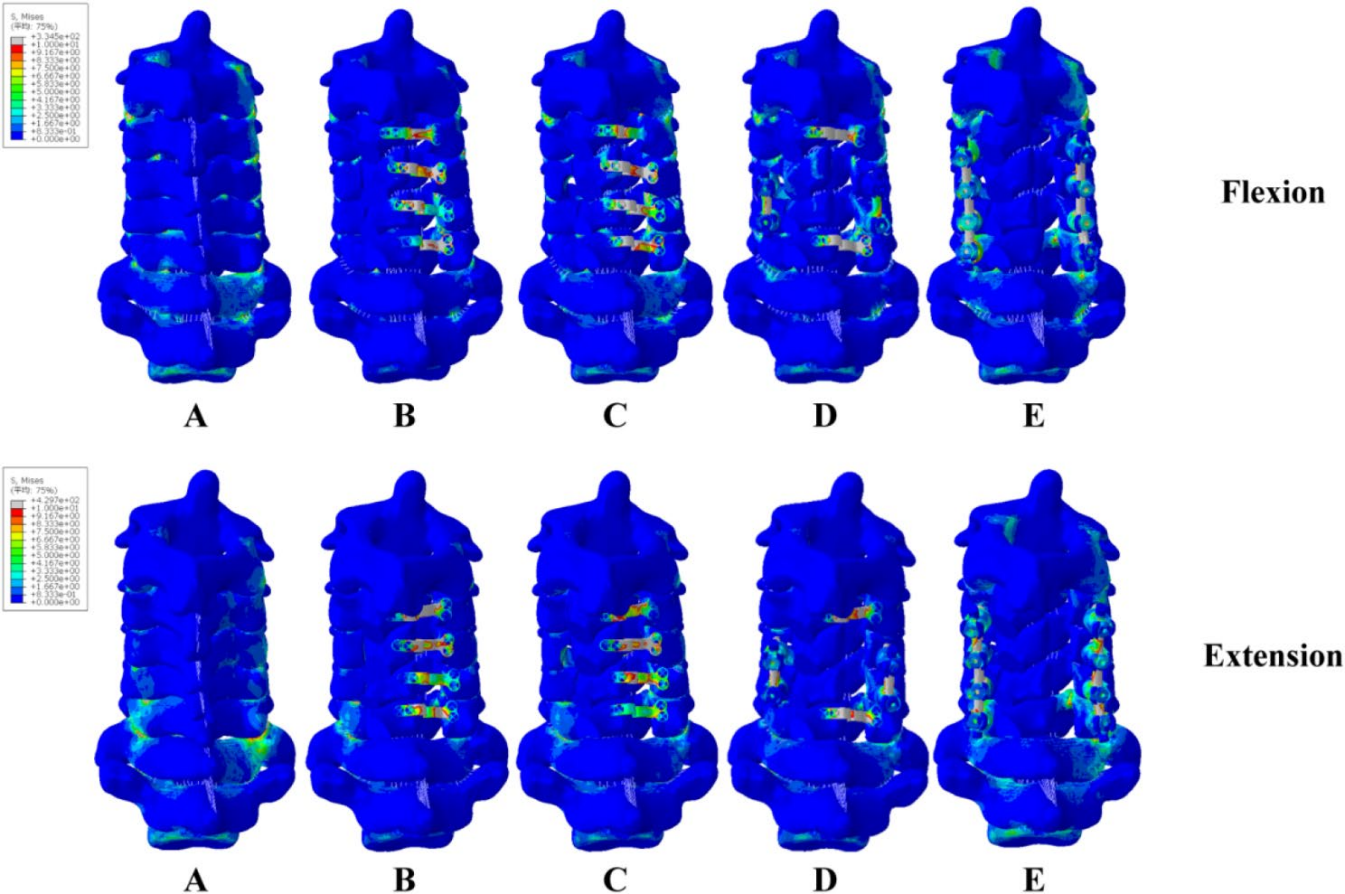
Stresses of five models in extension and flexion

**Fig. 9:**
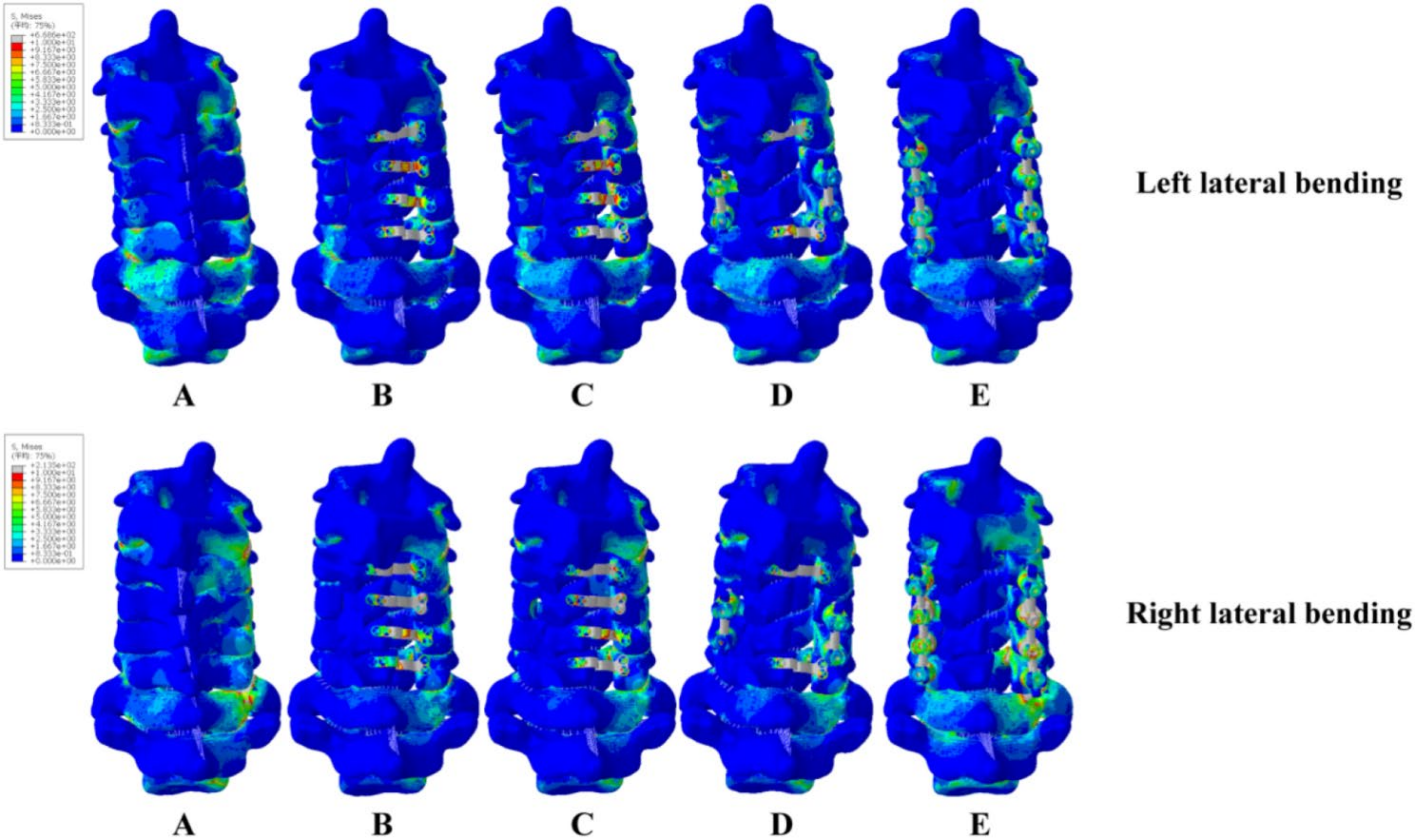
Stresses of five models in left and right lateral bending

**Fig. 10:**
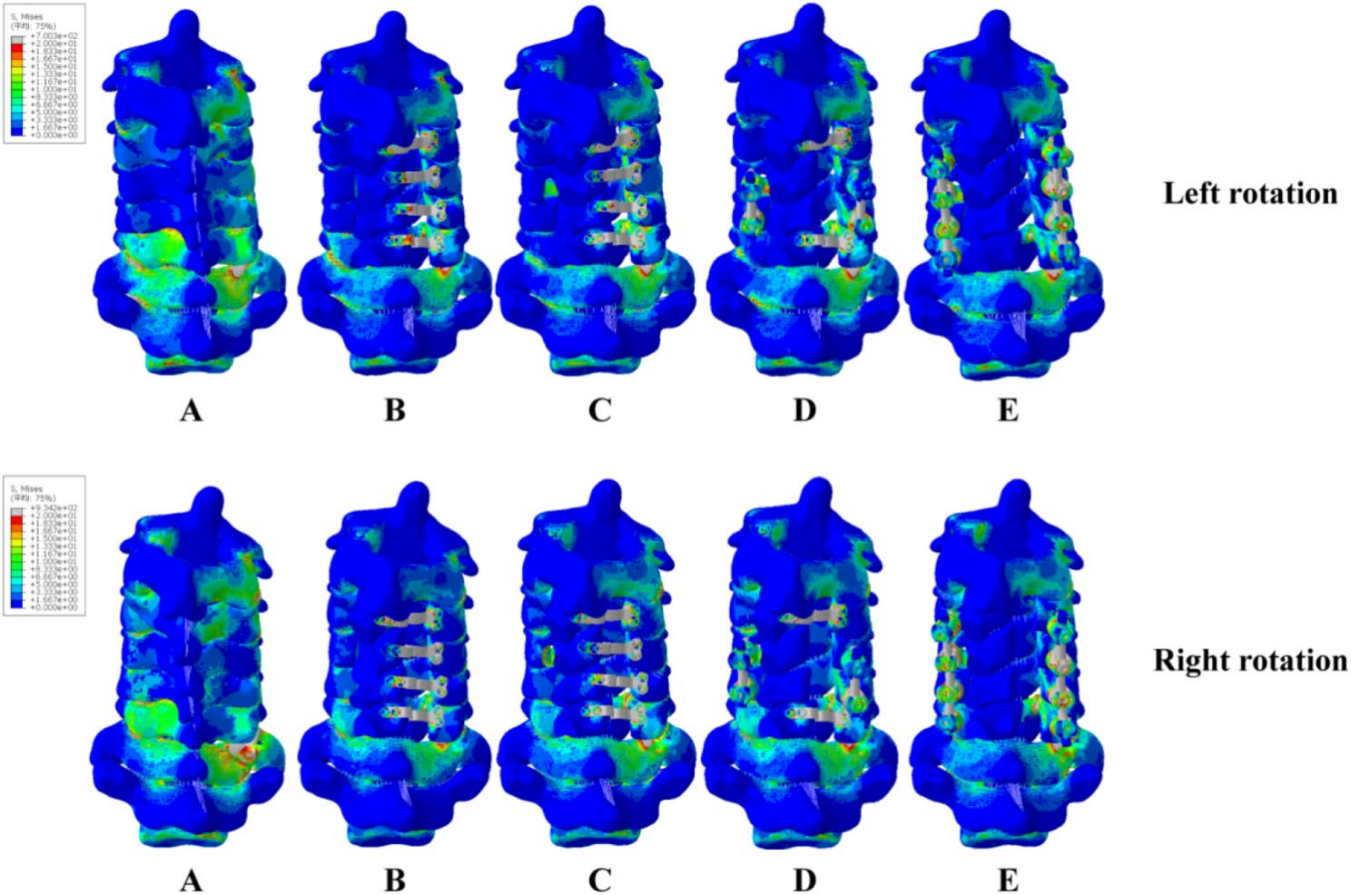
Stresses of five models in left and right rotation

Figures 8, 9 and 10 showed the stress distribution under six different motion states in five models. The stress in Model B was primarily concentrated at the fixing nails. In Model C, the stress in support plate increased. In Model D, the stress in support plate increased even more. Besides, the stress in C4/5’s connecting rods of model D was also higher. The stress in Model E’s screws and connecting rods were significantly increased. In flexion, model B had the greatest stress at the 2nd screw on the left side of C3. Model C had the greatest stress in the support plate of C4. Model D had the greatest stress concentrated at the screw on the right side of C5, and model E had the greatest concentration of stress at the left screw of C6. In extension, the maximum stress in Model B was still concentrated at the 2nd screw on the left side of C3. The stress was highest at the left joint capsule of C5/6 in Model C and Model D. The maximum stress in model E was located at the right side of the joint capsule in C7/T1. In left lateral bending, Model B’s right lower fixation screw of C3 had the highest stress, followed by peak stress at the left joint capsule of C7/T1 in Model C. The peak stress in Model D located at the left joint capsule of C3/4. The peak stress in Model E was at the right connecting rod of C6. The peak stress in Model B was located in the same place as in left lateral bending in the right lateral bending position, and the peak stress in Model C was located at the right joint capsule of C3/4. The peak stress in Model D was at the right screw of C5, and the peak stress in Model E was in the same location as in left lateral bending. In left rotation state, the peak stress of Model B was focused on the second fixation screw on the left side of C3. The peak stress of Models C and D was concentrated on the C6 support plate. The peak stress of Model E was concentrated on the right side of C3. In the right rotation condition, the screw at the right head end of C3 for Model B experienced the highest stress. Model C showed the same maximum stress location as the left rotation state. The peak stress in Model was at the screw on the right side of C5. The peak stress in Model E was at the screw on the right side of C4.

## Discussion

CSM accounts for 12–30% of all cases of cervical spondylosis [14–16]. EODL, an internationally recognized major surgical procedure for the treatment of CSM. C5 palsy is a unique complication after cervical decompression surgery with a statistical incidence of 7% [17–19]. One hypothesis thought that C5 nerve root palsy is mainly associated with nerve root tethering effect due to posterior displacement of the spinal cord after EODL [20]. This is mainly determined by the anatomical characteristics of the cervical spine. The C4/5 segment is located at the apex of the physiological anterior convexity of the cervical spine, so the C5 nerve root is shorter than the other nerve roots and has poor freedom. Moreover, the spinal cord of the C5 segment has the largest distance of posterior displacement after surgery, and the excessive traction causes nerve root tethering, which in turn leads to the C5 palsy. Machino et al. previously identified C5 palsy as deltoideus muscle paralysis, with or without biceps involvement, and no other loss of muscle power [21]. Researchers have discovered, however, that patients with C5 palsy not only have motor deficiencies, in severe cases, sensory paralysis as well, which leads to excruciating pain. Some researchers revealed that bilateral decompression of the C4/5 root canal successfully prevents the development of C5 nerve root palsy following surgery [4.^7^.^22^].

However, decompression of the nerve root canal can damage the articular process [23], resulting in decreased cervical spine stability, accelerating cervical degeneration and osteophyte formation, and affecting spinal cord function recovery. Short-segmented lateral mass screw fixation can be a good solution to the problem and increase the stability of the cervical spine after surgery, thus reducing the incidence of incisive cervical degeneration. There are few studies comparing different internal fixation modalities of EODL for the treatment of CSM using the FE method. We investigated the stability and stresses of four internal fixation devices by comparing their biomechanical features to facilitate clinical comprehension and selection.

The ROM of Model C in this experiment was significantly larger than that of Models A and B, as shown in Fig. 6, indicating that nerve root canal decompression of C4/5 would result in a reduction in spinal stability. The ROM of Model E was also worse than that of Models C and D, indicating that EODL with bilateral C4/5 foraminotomies and C3-6 lateral mass screw fixation would greatly improve spinal stability. However, it would also drastically reduce the cervical spine’s movement, which would have a greater effect on the patients’ daily lives. The ROM of Model D was significantly reduced compared to that of Model C. However, the ranges of motion of Model D and Model B in extension and flexion were not significantly different. This suggestted that the use of lateral mass screw fixation following decompression of the C4/5 nerve root canal contributes to increase spinal stability and has no negative impact on the patient’s day-to-day activities. Model D also maintains the ROM of the cervical vertebrae and ensures the stability of the cervical vertebrae, which is consistent with our clinical experience. C5/6 and C6/7, however, were more mobile in Model D than in Models A and B, which may enhance the likelihood of C5/6 and C6/7 degeneration.

The force on the support plate of Model C following foraminotomy was much greater than that of Model B, showing the strength of the internal fixation system in Figs. 8, 9 and 10. Increased stress over a long period of time can easily lead to metal fatigue, which in turn can lead to fracture and loosening of the internal fixation. The risk of fracture for the support plates of the relevant segments would increase because the stress on the C3 and C6 support plates of Model D was the greatest in forward flexion and greatly increased in posterior extension. The stress on the support plate during lateral bending was substantially greater in Model D than that in Model C.

The stress on the connecting rods was greater in Model D. This implied that the stress on the internal fixation system during lateral bending is significantly greater following C4/5 short segment lateral mass screw fixation, which indicated higher risk of fartigue fracture in the future. The stress on the internal fixation system increased following foraminotomy in both left and right rotation, which is most likely related to the decreased cervical spine stability after foraminotomy. Increased stress at the support plate, fixation nails and lateral mass screws causes screw loosening and fracture, which should be noted clinically. Increased stress at the support plate and connecting rods maybe fracture the corresponding internal fixation [24]. Increased stress at the joint capsule increases the risk of degeneration of the corresponding segment, which increases the likelihood of lesion development in the corresponding segmen. Thus, activities in the corresponding position should be limited.

Our study supports the nerve root tethering theory, but it is important to recognize that C5 palsy is a multifactorial complication and future research is needed to elucidate the complete mechanism. While our study suggests promising biomechanical effects, clinical studies would be required to confirm these effects in a patient population. Particularly, instead of stating a definitive reduction in C5 palsy, our findings suggest a potential biomechanical benefit that could be associated with a lower incidence of C5 palsy. Therefore, case series or prospective clinical trials are needed to evaluate the actual postoperative incidence of C5 palsy and the effectiveness of EODL with C4/5 short segment fusion in preventing it.

There were certain restrictions in the current therotical study. First, the finite element model used in this work was created using data from just one patient, which makes it impossible to avoid the variations in findings caused by individual morphological variations and thereby necessitates the use of additional samples for revalidation [25]. Second, because it is challenging to accurately depict anatomical details and the properties of the material, it can not simulate the real condition completely, the three-dimensional finite element model developed in this study has several drawbacks [26]. For instance, the effect of annular fibers in the annulus fibrosus was not taken into account when building the intervertebral disc in our model, which was only designed as a linear elastic material. Thirdly, the specifics of the patient’s long-term postoperative state could not be ascertained because the impact of internal fixation on nearby segmental degeneration was not taken into account [27]. Finally, the degree of cervical disc degeneration and cervical curvature are additional factors that affect the actual results of surgery and must be examined in vitro tests and clinical investigations [28].

## Conclusion

Compared to C4/5 lateral mass screw fixation, C3-6 lateral mass screw fixation significantly decreased cervical spine motion and has a higher detrimental effect on the patient’s daily life. EODL combined with bilateral C4/5 foraminotomy and short-segment lateral mass screw fixation can effectively relieve compression, maintain the stability of the spine, have minimal effects on the cervical spine ROM in the extension and flexion state, and may improve postoperative C5 palsy. Although more clinical studies are needed to confirm these findings, it may be a promising treatment option for CSM and prevention of postoperative C5 palsy.

## Data Availability

No datasets were generated or analysed during the current study.
